# Permeability of the Perindopril Arginine under In Vitro Conditions across Caco-2 Monolayer and Biomimetic Phospholipid Membrane

**DOI:** 10.3390/molecules27072232

**Published:** 2022-03-30

**Authors:** Marta Kus, Klaudia Gorniak, Piotr Czaklosz, Anna Olejnik, Paulina Skupin-Mrugalska, Izabela Ibragimow, Hanna Piotrowska-Kempisty

**Affiliations:** 1 Department of Toxicology, Poznan University of Medical Sciences, 30 Dojazd St., 60-631 Poznan, Poland; 84499@student.ump.edu.pl; 2 Research and Development Department of Ethifarm, Ethifarm Manufacturing Plant, 9 Stefana Zeromskiego St., 60-544 Poznan, Poland; klaudia.gorniak@ethifarm.pl (K.G.); business.development@ethifarm.pl (P.C.); izabela.ibragimow@ethifarm.pl (I.I.); 3 Department of Biotechnology and Food Microbiology, Poznan University of Life Sciences, 48 Wojska Poskiego St., 60-627 Poznan, Poland; 4 Department of Inorganic and Analytical Chemistry, Poznan University of Medical Sciences, Grunwaldzka 6 St., 60-780 Poznan, Poland; psmrugalska@ump.edu.pl; 5 Department of Basic and Preclinical Sciences, Institute of Veterinary Medicine, Nicolaus Copernicus University in Toruń, 7 Gagarina St., 87-100 Torun, Poland

**Keywords:** perindopril arginine, permeability, Caco-2 monolayer, PermeaPad^®^ 96-well plate, simple diffusion, intestinal absorption

## Abstract

Perindopril arginine (PA) as an angiotensin-converting enzyme (ACE) inhibitor is widely used in cardiovascular diseases, especially in systemic hypertension and heart failure. Although the pharmacokinetics of PA are well documented, there is no available detailed data on its permeation in in vitro conditions. The present study aimed to assess the transport of PA across both biological membranes and artificial biomimetic ones. For the determination of PA transport, the Caco-2 cell line was selected as a reliable in vitro model of gastrointestinal biological barriers. Additionally, a novel 96-well plate with phospholipid membrane PermeaPad was used to evaluate the transport of PA by passive diffusion. We confirmed that PA is relatively poorly permeable across the Caco-2 monolayer. The permeability results obtained from the non-cell-based model demonstrated higher transport of PA as compared to that of Caco-2. Thus, PA transport across the biological membranes might be suggested to be regulated by the membrane transporters.

## 1. Introduction

Oral administration of drugs is the most frequent dosage form due to the patient convenience and the cost-effective manufacturing. The bioavailability of orally administered drugs is largely dependent on an active pharmaceutical ingredient (API) absorption across the biological membrane of the gastrointestinal tract (GIT). Human studies provide direct knowledge regarding drug bioavailability; however, they are costly and time-consuming. Thus, in vitro absorption models play a prominent role in the development process of novel drugs’ dosage forms and selection of the compounds with advantageous pharmaceutical properties [1]. In particular, the in vitro cell models for permeability studies are widespread, since they mimic the intestinal epithelium monolayer and transport mechanisms [2].

The human colon adenocarcinoma cell line Caco-2 [3] is the most common cell-based system to predict intestinal drug permeation in in vitro conditions. The Caco-2 cell line has been approved by the regulatory institutions Food and Drug Administration (FDA) and European Medicines Agency (EMA) as a reliable in vitro model for the prediction of human oral bioavailability [4,5]. Caco-2 cells spontaneously differentiate after confluence, and then they exhibit morphological and functional characteristics of small intestinal enterocytes. The differentiated Caco-2 monolayer is characterized by the tight junctions between the cylindrical cells with the apical brush borders, as found in the small intestine [6]. The Caco-2 monolayer has been shown to present many membrane transporters, including sugar (GLUT1, GLUT2, GLUT3, GLUT5) [7,8] and peptide (PEPT1 and HPT1) ones [9], ion channels (Na+/H+ transporter) [10] and P-gp efflux transporter [11]. However, the Caco-2 cells maintained in different laboratories exhibit large variability and different transport features due to culture conditions and the passage number [12].

The non-cell-based models are usually applied for rapid permeability screening of drug substances. The artificial Permeapad^®^ membrane was developed in 2015 by di Cagno and co-workers [13] and as a 96-well plate from innoME is used for high-throughput permeability studies [14]. In contrast to the widely used Parallel artificial membrane permeability assay (PAMPA), it is constructed of dry phospholipids (soy phosphatidylcholine, S-100) deposited between two cellulose-hydrate membranes without filter support [13,14,15]. Phospholipids swell with the aqueous media contact and form myelin-like structures, thus mimicking a biological membrane [14,15]. However, Permeapad^®^ is an artificial membrane, and in contrast to the cell models like the Caco-2 monolayer, it can be used for only simple diffusion permeability profiling. Nevertheless, it is a favorable model for the compounds’ permeability screening due to the high resistance to environmental pH changes as well as the cost and time effectiveness [13].

Perindopril arginine (PA) is a prodrug that, after oral administration, is hydrolyzed by the hepatic esterase to the active diacid metabolite perindoprilat [16]. PA, as an angiotensin converting enzyme (ACE) inhibitor, is widely used in cardiovascular diseases, especially in systemic hypertension [17] and heart failure [18]. PA comes in the form of tablets in doses of 2 mg, 4 mg or 8 mg and can be used alone or in combination with other agents [19]. PA is in the form of two salts—erbumine (synonym tert-butylamine) and arginine, indicating the same action. However, PA as arginine salt exhibits greater shelf life and stability under conditions of high temperatures and relative humidity [20]. After oral administration, PA is rapidly absorbed with peak plasma concentrations and is completed within approximately 1 h [21]. About 30–50% of the perindopril is metabolized into perindoprilat during the first pass of the liver. The absolute oral bioavailability of PA is 75% and the average bioavailability of its active metabolite is about 25% [22]. Perindoprilat is detectable in the plasma within 30 min after administration [23], and peak plasma concentrations are attained within 3 to 7 h [22]. The mean half-life of PA is about 0.8 to 1.0 h [22], while perindoprilat is 5–11 h [23].

Although the pharmacokinetics of PA are well documented, there is no available detailed data on its permeation across the biological membranes or biomimetic barriers in in vitro conditions. PA is considered to belong to the group III of the Biopharmaceutical Classification System (BCS) [23] and is characterized by low permeability [4,5]. However, detailed parameters determining its permeability in in vitro conditions, such as apparent permeability coefficients (P_app_), are not available. Hence, the present study aimed to determine the transport of PA across the Caco-2 monolayer. The obtained results were compared with those of the cell-free permeation barrier PermeaPad^®^. In both of the models used, we performed the permeability assays for caffeine as a highly permeable substance. Since our previously published data showed a high permeation of Naftidrofuryl oxalate (NF) across the Caco-2 monolayer [24], we also applied this drug for the transport study through the biomimetic membrane Permeapad.

## 2. Results

### 2.1. Effect of PA on Caco-2 Cells’ Viablility

**Figure 1:**
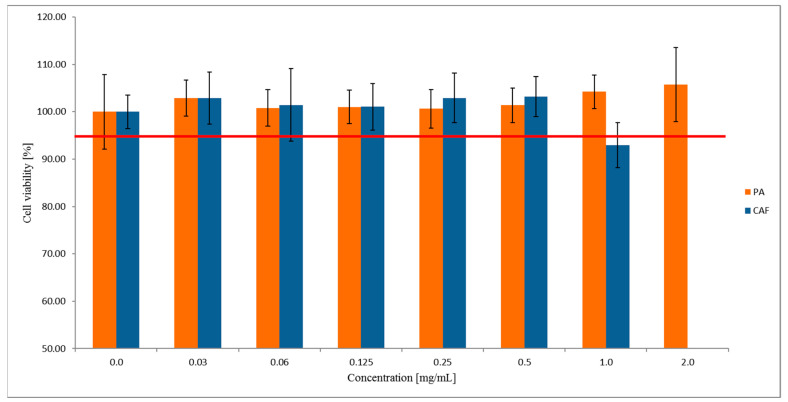
The viability of the Caco-2 cell line treated with PA and CAF. Results of the three independent replicates are presented as the mean ± SD. Acceptance criterion for the cell viability exposed for different compounds concentration has been established as 95% (red line).

The concentrations of PA for the permeability tests were determined using the MTT assay as a standard rapid cytotoxicity test. For this purpose, Caco-2 cells were exposed for two hours to PA at the concentration range of 0.0–2.0 mg/mL. As the reference standard, CAF was used in the concentration range from 0.0 mg/mL to 1.0 mg/mL. The MTT results (Figure 1) showed that PA was not cytotoxic to the Caco-2 cell line in all concentrations tested. Concomitantly, CAF at the concentration of 1.0 mg/mL was observed to decrease the viability of Caco-2 cells to ~90%. Since only compounds’ concentrations with the cell viability of at least 95% are commonly accepted for the permeability assays, the cytotoxicity study for the higher concentrations of CAF was not continued.

Based on the MTT results, for further permeability studies across the Caco-2 monolayer, we used the two highest concentrations of PA 2.0 mg/mL and 1.0 mg/mL, and 0.5 mg/mL concentration for CAF.

### 2.2. Permeability Studies across the Caco-2 Monolayer

**Figure 2:**
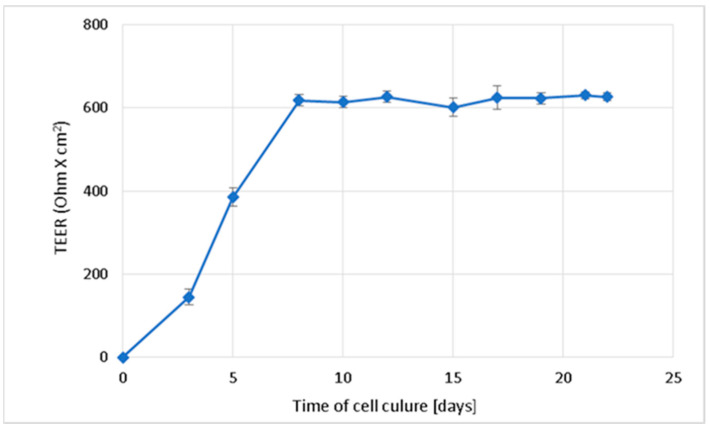
Change in the transepithelial electrical resistance (TEER) over time during Caco-2 cell culture on membranes directed towards the formation of the integral intestinal epithelium. Results of the three independent replicates are presented as the mean ± SD.

The integrity of the Caco-2 monolayer during the 22-day culture was monitored by measuring the transepithelial electrical resistance (TEER). The permeability tests of PA and CAF were performed only in the Caco-2 monolayers with TEER values beyond 600 Ohm/cm^2^ (Figure 2). The obtained results indicate sufficient differentiation of Caco-2 cells and their suitability for further studies on the permeability of the compounds.

**Figure 3:**
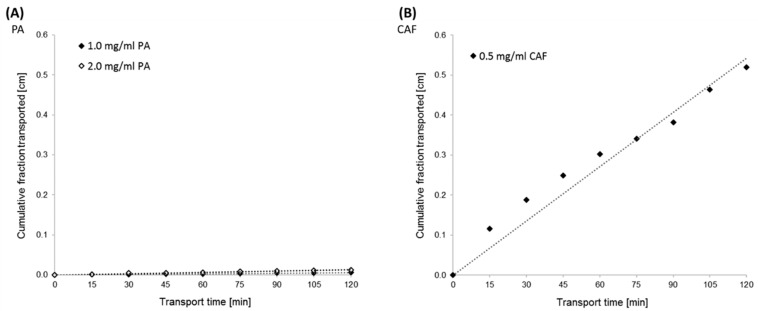
Kinetics curves of perindopril arginine (PA) (**A**) and caffeine (CAF) (**B**) transport across the Caco-2 monolayer. The experimentally (◊) and theoretically (······) determined “cumulative fraction transported” of the compounds versus time transport. PA transport was analyzed at the initial concentrations of 2.0 mg/mL and 1.0 mg/mL. The initial concentration of CAF was established at 0.5 mg/mL.

The permeability study of PA and CAF across the Caco-2 monolayer was performed from the basolateral to the apical compartment (bottom to top) for 2 h. Based on HPLC data, the PA and CAF cumulative fraction transported (CFT) (Figure 3) and the apparent permeability coefficient (P_app_) values (Table 1) were evaluated.

The results obtained from transport experiments showed that the permeability of PA across the Caco-2 monolayer is relatively poor. Permeability coefficients estimated for the two concentrations of PA were lower than 2 × 10^−6^ cm/s. The P_app_ values were calculated at 1.94 × 10^−6^ cm/s and 0.72 × 10^−6^ cm/s for PA at the concentrations of 2.0 mg/mL and 1.0 mg/mL, respectively (Table 1). Concomitantly, the high permeability of CAF across the Caco-2 monolayer was confirmed. CFT in the receiver compartment of the Caco-2 experimental model increased linearly with time for all tested compounds (Figure 3).

### 2.3. Permeability Studies across PermeaPad Membrane

**Figure 4:**
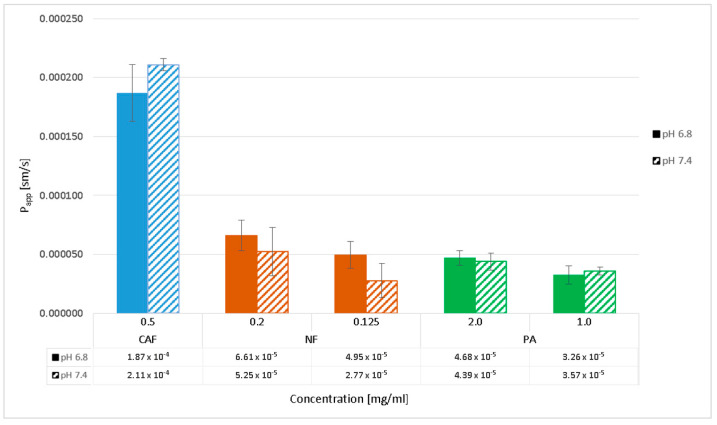
Apparent permeability coefficients (P_app_) of perindopril arginine (PA), caffeine (CAF) and naftidrofuryl oxalate (NF) determined in the PermeaPad barrier in pH 6.8 and 7.4. Compounds’ transports were analyzed at the initial concentrations of 2.0 mg/mL and 1.5 mg/mL for PA, 0.5 mg/mL for CAF, and 0.2 mg/mL and 0.125 mg/mL for NF. Results are reported as an average and standard deviation (*n* = 3).

**Figure 5:**
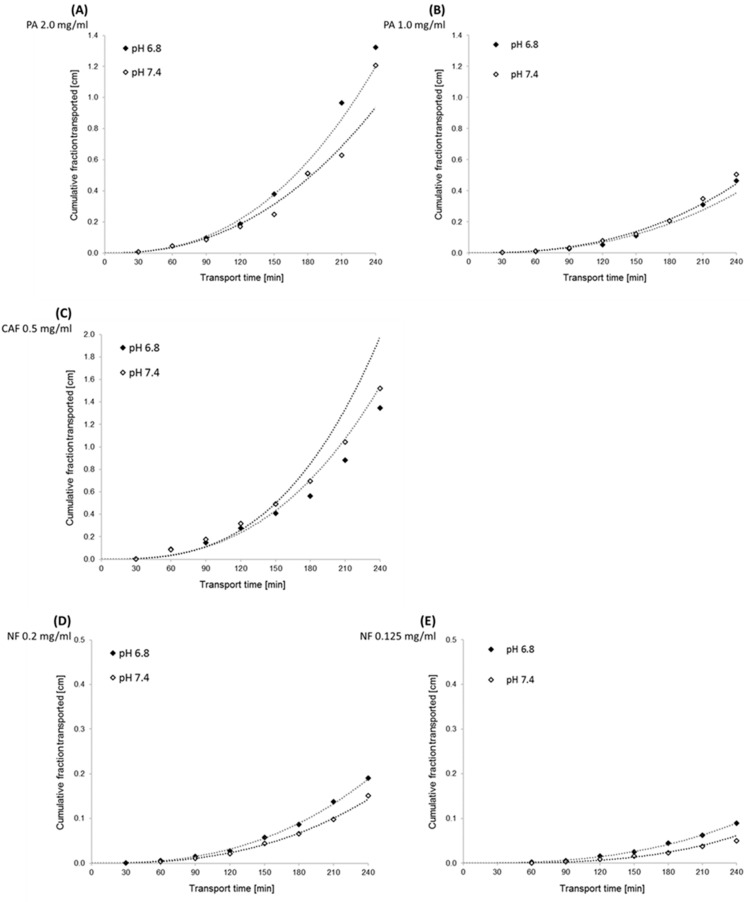
Kinetics curves of perindopril arginine (PA), caffeine (CAF) and naftidrofuryl oxalate (NF) transport across the PermeaPad barrier. The experimentally (◊) and theoretically (······) determined “cumulative fraction transported” of compounds versus time transport. Compounds’ concentrations in donor compartments were established at 2.0 mg/mL (**A**) and 1.5 mg/mL (**B**) for PA, 0.5 mg/mL for CAF (**C**), and 0.2 mg/mL (**D**) and 0.125 mg/mL (**E**) for NF.

Transport across the PermeaPad barrier was performed in media at two different pH levels, 7.4 and 6.8, to evaluate the effect of pH change on the permeability of compounds. The permeability of PA and CAF was analyzed at the same concentrations as for transport through the Caco-2 monolayer. For additional control of the PermeaPad barrier, we performed a permeability test of Naftidrofuryl oxalate (NF) in two concentrations 0.125 mg/mL and 0.2 mg/mL. During the 4-h transport, samples of each compound were taken every 30 min and then analyzed by HPLC. The results of apparent permeability and CFT for PA, CAF and NF are shown in Figure 4 and Figure 5, respectively.

The results obtained from the transport experiments showed that the rate of transport of the compounds across the PermeaPad barrier is inversely proportional to the size of the molecules, concentration dependent, and pH-medium independent. The highest transport by passive diffusion was observed for the smallest compound, CAF (MW = 194.2), in both mediums at pH 6.8 and 7.4. The lowest permeability was observed for PA as the largest molecule (MW = 542.7), despite the highest concentrations used (2.0 mg/mL and 1.0 mg/mL). We observed a lower permeability of NF compared to CAF; however, the concentrations of NF (0.2 mg/mL and 1.0 mg/mL) were about 2.5 and 4.0 times lower than CAF. Hence, it can be assumed that the permeability of NF (MW = 473.6) across the PermeaPad barrier is relatively high (Figure 4).

Additionally, we showed that different mediums at pH 6.8 and 7.4 did not affect PA permeability. We observed slightly lower permeability of NF in medium at pH 7.4, as compared to that of pH 6.8. However, the statistical evaluation of the transport of all compounds across the PermeaPad barrier (Student t-test) showed no significant differences in the permeability of the substances relative to the two different media at pH 6.8 and 7.4 (*t*-test, *p* > 0.05).

Cumulative fraction transported (CFT) in the receiver compartment of the PermeaPad model increased non-linearly with time for all tested compounds. The transport kinetics of NF and PA were dependent on the initial concentration of the compound (Figure 5).

## 3. Discussion

The in vitro permeation system plays a significant role in predicting intestinal absorption of drug substances and transepithelial transport. PA has been characterized the by high solubility and low permeability [23]. However, to the best of the authors’ knowledge, there is no available detailed data concerning the transport of PA through biological membranes and/or biomimetic barriers. Thus, in this study, we investigated the permeability of PA in two in vitro permeation systems; the widely used Caco-2 and the new biomimetic PermeaPad ones.

To confirm the suitability of both permeation models used in the present study, the permeability test of CAF was performed. CAF is a model substance for which high permeability in the Caco-2 cell line (P_app_ = ~50 × 10^−6^ cm/s; concentration 0.06 mg/mL) [24,25], and in the PermeaPad barrier (P_app_ = 2.4 × 10^−5^ cm/s; concentration 5 mM) [13] has been well documented. The choice of PA and CAF concentrations to the permeability assays across the Caco-2 monolayer was based on the results of the MTT assay. The cytotoxicity of PA in Caco-2 cells was assessed in the concentration ranges of the compound from 0.0 mg/mL to 2.0 mg/mL. No effect of PA concentration on the viability of the Caco-2 cell line was observed. Therefore, the transport of PA was investigated at the two highest concentrations (1.0 mg/mL and 2.0 mg/mL) to assess the concentration substance permeability relationship.

For the cytotoxicity assay, CAF was used in the concentration range from 0.0 mg/mL to 1.0 mg/mL. Since the higher concentration of CAF (1.0 mg/mL) decreased the Caco-2 cells viability to 93%, we used 0.5 mg/mL dose in the permeability assays.

As an additional control of permeation across the PermeaPad biomimetic membrane, NF transport was performed. Our previously published results showed high permeability of NF through the Caco-2 monolayer at the concentrations 0.125 mg/mL (P_app_ = 84.1 × 10^−6^ cm/s) and 0.2 mg/mL (P_app_ = 78.8 × 10^−6^ cm/s) [26]. Hence, the same concentrations of NF were applied in the PermeaPad barrier for a direct comparison with the data obtained in the Caco-2 cell model.

Due to easy sampling, the transport of all compounds in the two permeation systems was carried out from the lower to the upper compartment (bottom to top). The permeability assays across the PermeaPad barrier were performed in media at two different pH levels, 6.8 and 7.4, mimicking the physiological conditions of different parts of the small intestine (duodenum pH 6.8, distal part pH 7.4). However, due to the limitations of maintaining the cell culture at pH 6.8, the transepithelial transport through the Caco-2 monolayer was performed at a more physiological pH, 7.4. Transport study of each compound across the PermeaPad barrier was performed at room temperature (RT), while transport assay through Caco-2 was carried out at 37 °C due to the maintenance of cell culture. Although simple diffusion is temperature dependent, in the present study only RT was used, according to the manufacturer’s protocol of the PermeaPad barrier [27].

According to the literature data, the highly permeable substances are characterized by P_app_ > 10 × 10^−6^ cm/s. Concomitantly, when P_app_ is lower than 1 × 10^−6^ cm/s, the substance is considered poorly permeable [28,29]. The suitability of the Caco-2 cell monolayer for permeability test was confirmed in TEER measurement. Moreover, since we observed high transport of CAF through both the Caco-2 monolayer and the PermeaPad membrane, the usefulness of the permeation models used was confirmed. However, the transport of CAF in the cell-based permeation model was shown to be lower than that by passive diffusion. Statistically significant differences (*t*-test, *p* < 0.05) have been observed between the permeability of CAF across the Caco-2 monolayer and the PermeaPad barrier. Additionally, the P_app_ values for CAF transported across the PermeaPad barrier in different media were quite similar and no significant difference (*p* > 0.05) was measured for pH 6.8, and 7.4. Caffeine is a weak base with a pKa value of 14.0 and its neutral form is likely predominant in a medium at a pH of 6.8 and 7.4. However, as a small and sufficiently lipophilic molecule (log-P = 0.07), it is a high permeable substance across the PermeaPad phospholipid barrier by passive diffusion.

Since the estimated P_app_ values of PA permeation across the Caco-2 monolayer in both tested concentrations were shown to be lower than 1 × 10^−6^ cm/s, we confirmed its poor permeability. For our study, we used 2.0 mg/mL and 1.0 mg/mL PA concentrations, and the dependence of PA permeability on its concentration was observed. However, we demonstrated the relatively high transport of PA by passive diffusion using the PermeaPad barrier as compared to its permeability in Caco-2 cells. PA is the largest compound among those used in our study and despite the highest concentration used, PA permeability through the PermeaPad barrier was shown to be lower than the high permeability substances CAF and NF. Additionally, we observed no significant differences (*t*-test; *p* > 0.05) in PA permeability in media at pH values of 6.8 and 7.4 for both concentrations tested. PA is an acid with pKa values of 3.17 and 5.67, and its ionized in the media of pH 6.8 and 7.4. Hence, due to a slight pH difference in the media used, we did not observe significant differences in its permeability. PA is compound with a log-P of 0.65, and its lipophilicity is optimal for ensuring good permeability and good solubility. However, the relatively low permeability of PA by passive diffusion, in contrast to NF and CAF, is due to the high molecular weight and ionization of the compound. The fact that PA is poorly absorbed through Caco-2 monolayers (P_app_ 1.94 × 10^−6^ cm/s for 2.0 mg/mL, and 0.72 × 10^−6^ cm/s for 1.0 mg/mL) and not on the Permeapad membrane (P_app_ 43.9 × 10^−6^ cm/s for 2.0 mg/mL, and 35.7 × 10^−6^ cm/s for 1.0 mg/mL) is likely related to the membrane transporters specific for the Caco-2 cell line. According to the literature data, PA has relatively poor interaction with the intestinal peptide transporters (PEPT1, PEPT2) [30]. Knutter et al. suggested that other membrane carriers, especially the organic anion-transporting family members (SLC21 and SLC22) should be considered in relation to the membrane transport of ACE inhibitors [31]. Hence, further studies on the transepithelial transport mechanisms of PA in the Caco-2 cell model need to be performed.

In the present study, we showed that the permeability of NF through the PermeaPad membrane is high since the P_app_ values in both tested concentrations were larger than 10 × 10^−6^ cm/s. Additionally, we observed that the permeability of NF was concentration dependent. Our results showed slightly lower P_app_ values for both concentrations of NF in medium at pH 7.4 in comparison to those of pH 6.8 (*t*-test; *p* > 0.05). NF is a compound with pKa values of 4.1 and in a medium at pH 7.4 it is likely more ionized than at pH 6.8. For the permeability assay, we used 0.2 mg/mL and 0.125 mg/mL NF concentrations, which were approximately three and four times lower than CAF concentration (0.5 mg/mL), respectively. Therefore, it might be suggested that the permeability of NF by simple diffusion is likely as high as the good permeability of CAF, which is a much smaller compound. Additionally, we showed that transport of NF through the PermeaPad barrier by simple diffusion was slightly higher than across the Caco-2 monolayer. We observed a statistically significant difference (*t*-test, *p* < 0.05) between permeability of NF across the Caco-2 monolayer and the PermeaPad barrier for only 2.0 mg/mL concentration.

## 4. Materials and Methods

### 4.1. Permeability Studies Employing PermeaPad Membrane

#### 4.1.1. Chemical Reagents and References

Acetonitrile (ACN) with chromatographic grade from VWR Chemical (Fontenay-sous-Bois, France) was used for the HPLC assay. Other reagents with analytical grade were obtained from Chempur (Piekary Slaskie, Poland): hydrochloric acid (HCl), disodium hydrogen phosphate (Na_2_HPO_4_), potassium dihydrogenphosphate (99%, KH_2_PO_4_) ammonium acetate (CH_3_CO_2_NH_4_), sodium acetate (CH_3_COONa), glacial acetic acid (CH_3_COOH), orthophosphoric acid (85%; H_3_PO_4_) and timethylamine (TEA). Ultrapure water (H_2_O) used for all experiments was obtained from the MilliQ purification system (Direct-Q3 Water Purification System, Merck Group, Darmstadt, Germany).

#### 4.1.2. In Vitro Analysis

Reagents from Sigma-Aldrich (Merck Group, Darmstadt, Germany) were used for prepared cell culture medium: Dulbecco’s Modified Eagle’s Medium with low glucose (DMEM), fetal bovine serum (FBS), 1% nonessential amino acids (MEM NON), and 2 mM L-glutamine, 100 U/mL penicillin and 0.1 mg/mL streptomycin solution. Other reagents for in vitro testing were obtained from Sigma Aldrich (Merck Group, Darmstadt, Germany): Trypsin-EDTA solution, Hank’s Balanced Salt Solution (HBSS). The MTT (3-(4,5-dimethyl-2-thiazolyl)-2,5-diphenyl-2H-tetrazolium bromide) test from Sigma-Aldrich (Merck Group, Darmstadt, Germany) was used for cytotoxicity analysis. Dimethyl sulfoxide (DMSO) was obtained from POCH-Avantor (Gliwice, Poland). Phosphate-buffered saline (PBS) with pH 6.8 and 7.4 from Sigma-Aldrich (Merck Group, Darmstadt, Germany) was used for the permeability study with the PermeaPad barrier. The pH for each solution was controlled before the addition of the drug substance and after its addition. The pH was adjusted using NaOH or HCl if necessary (measured by pH meter sensIONTM PH31; HACH Lange, Wroclaw, Poland).

Plastic tissue culture vessels were purchased from Corning-Life Science (Durham, NC, USA). For the permeability study across the Caco-2 monolayer, a cell culture insert from Millipore (Merck Group, Darmstadt, Germany) was used.

#### 4.1.3. Compounds

PA was obtained from Ethifarm (Ethifarm, Poznan, Poland). Reference substances CAF and NF were obtained from Sigma Aldrich (Merck Group, Darmstadt, Germany). Chemical properties of all compounds are shown in Table 2.

### 4.2. Caco-2 Cell Culture

The human colon cancer cell line Caco-2 (HTB-37TM) was purchased from the American Type Culture Collection (Manassas, Virginia, USA). Cells were grown in phenol red-free DMEM supplemented with 20% FBS, 1% MEM NON and 2 mM L-glutamine, 100 U/mL penicillin, and 0.1 mg/mL streptomycin solution. The cells’ maintenance was carried out at 37 °C in a humidified atmosphere containing 5% CO_2_ and 95% air. The Caco-2 cell line was grown in a 75 cm2 tissue culture flask, and the DMEM cell culture medium was changed every other day (48 h). The cell culture was passaged using Trypsin-EDTA solution after cells reached approximately 80% confluence (estimated by Light Microscopes Promovert, Zeiss Group, Oberkochen, Germany).

### 4.3. Cytotoxicity Analysis

To assess the cytotoxic activity of PA and CAF, the confluent stock cultures Caco-2 were detached with Trypsin-EDTA solution and plated in 96-well plates at a density of 2 × 10^4^ cells/well (counting in the presence of trypan blue using Bűrker chamber) in 150 µL of DMEM cell culture medium. After leaving them overnight, different concentrations of analyzed substances in HBSS pH 7.4 were added.. Cells were exposed to the substances for 2 h (time of transport across monolayer) in an incubator under optimal culture conditions. After incubation, the solution was removed and the mixture of DMEM cell culture medium and MTT (5 mg/mL) was added. Cells were incubated for 2 h, and then the resulting formazan crystals were dissolved in DMSO. The absorbance was measured using an Elx-800 absorbance microplate reader (Biotek, Winooski, VT, USA) at wavelength 540 nm.

### 4.4. Permeability Testing across Caco-2 Monolayer

For the transport study, the Caco-2 cells were seeded at 4 × 10^5^ cells/well on the permeable cell culture inserts with a 0.4 µm pore diameter (Millipore-Merck Group, Darmstadt, Germany) placed in 6-well plates. The cells were cultured for 22 days in the incubator under optimal culture conditions (37 °C, 5% CO_2_, and 95% air) to their spontaneity differentiating. The integrity of the monolayer was tested by measuring the Trans Epithelial Electrical Resistance (TEER) every 48 h using a Millicel ERS-2 Voltohmmeter (Millipore-Merck Group, Darmstadt, Germany). The cells’ monolayer with an average TEER value > 600 Ohm × cm^2^ was considered to be suitable for permeability testing.

The permeability tests across the Caco-2 monolayer of substances were performed from basolateral to the apical compartment (B–A). Plates with cell culture inserts were incubated at 37 °C with shaking at 100 rpm for 2 h. Drug substances were dissolved in the transport buffer solution (HBSS with 25 mM HEPES, pH 7.4) and placed into a donor compartment. The solution volume in the apical and basolateral compartments was maintained at 2 mL and 4 mL, respectively. The samples (150 µL) from the acceptor compartment were taken at 15 min intervals and were replaced with fresh reheated (37 °C) buffer. The HPLC analysis described below was used to determine the concentration of the compound transported. Transport kinetics and the apparent permeability coefficient (P_app_) were calculated according to the protocols described by Tavelin et al. [33] and Hubatsch et al. [28].

### 4.5. Permeability Studying across PermeaPad 96-Well Plate

The Permeapad^®^ 96-well plate was purchased from InnoME GmbH (Espelkamp, Germany). The plate consists of a bottom plate, a screen plate with a lipid barrier and a lid. The surface area for permeation studying is 0.15 cm^2^. The permeability tests across a biomimetic barrier of drug substances were performed from the bottom to the top compartment. The compounds were dissolved in PBS with pH 6.8 or 7.4. The solution volume in the bottom and screen plate was maintained at 400 µL and 200 µL, respectively. The samples (50 μL) were taken every 30 min from the acceptor compartment for 4 h and replaced with an equal amount of fresh PBS at an appropriate pH. The permeation tests for all compounds were performed at room temperature (25 °C). HPLC methods were used to determine the concentration of the compound transported. The apparent permeability coefficient (P_app_) was calculated according to the protocol described by di Cagno et al. [13].

### 4.6. Quantification by HPLC

Concentrations of all compounds transported were determined by the reverse phase HPLC with UV-VIS detection. HPLC analysis was conducted on Waters UPLC Acquity H-class apparatus with Empower 3.0 software (Waters Corporation, Milford, MA, USA). The analytical method of CAF was based on the European Pharmacopoeia monograph [34], while NF was based on our previously published data [24]. The chromatographic parameters for PA were set as follows: column (C18: 4.6 mm × 75 mm; 3 µm); the liquid phase was a mixture of 60 volumes of phosphate buffer (pH = 2.8 with 85% *v*/*v* orthophosphoric acid) with 40 volumes of ACN and 0.2 volumes THF. The isocratic flow rate was set as 0.6 mL/min and the detection wavelength on λ = 215 nm. The retention time of the PA peak was about 3 min.

## 5. Conclusions

In the present study, we demonstrated very low permeability of PA across the Caco-2 monolayer. In contrast, PA transport through the PermeaPad biomimetic membrane was shown to be relatively high as compared to that of the cell-based model. Thus, we agree with the previous literature data that PA transport across the biological membranes might be regulated by the membrane transporters. However, further studies on the transepithelial transport mechanisms of PA in the Caco-2 cell model need to be performed.

## Figures and Tables

**Table 1:** Apparent permeability coefficients (P_app_) of perindopril arginine (PA) and caffeine (CAF) measured through Caco-2 monolayer. Results are reported as an average and standard deviation (*n* = 3).

Substance	Concentration (mg/mL)	P_app_ × 10^−6^ (cm/s)	Compound Permeability
PA	2.0	1.94 ± 0.96	low
PA	1.0	0.72 ± 0.04	low
CAF	0.5	126.5 ± 2.4	high

**Table 2:** Physicochemical properties of compounds.

Substance	Chemical Formula	Molecular Weight (MW)	pKa	logP	Reference
PA	C_25_H_46_N_6_O_7_	542.7	3.17 (strongest acid) 5.67 (strongest base)	0.65 0.56	internal data
CAF	C_8_H_46_N_6_O_7_	194.2	14	0.07	PubChem [32]
NF	C_26_H_35_NO_7_	473.6	4.1	1.98 4.85	internal data

## Data Availability

Not applicable.
